# Co-Occurrence of Fragile X Syndrome with a Second Genetic Condition: Three Independent Cases of Double Diagnosis

**DOI:** 10.3390/genes12121909

**Published:** 2021-11-27

**Authors:** Elisabetta Tabolacci, Maria Grazia Pomponi, Laura Remondini, Roberta Pietrobono, Daniela Orteschi, Veronica Nobile, Cecilia Pucci, Elisa Musto, Marika Pane, Eugenio M. Mercuri, Giovanni Neri, Maurizio Genuardi, Pietro Chiurazzi, Marcella Zollino

**Affiliations:** 1 Sezione di Medicina Genomica, Dipartimento Universitario Scienze della Vita e Sanità Pubblica, Università Cattolica del Sacro Cuore, Fondazione Policlinico Universitario A. Gemelli IRCCS, 00168 Rome, Italy; elisabetta.tabolacci@unicatt.it (E.T.); veronicanobile88@gmail.com (V.N.); cecilia.pucci@unicatt.it (C.P.); giovanni.neri43@gmail.com (G.N.); maurizio.genuardi@unicatt.it (M.G.); Marcella.Zollino@Unicatt.it (M.Z.); 2 UOC Genetica Medica, Fondazione Policlinico Universitario A. Gemelli IRCCS, 00168 Rome, Italy; mariagrazia.pomponi@policlinicogemelli.it (M.G.P.); laura.remondini@policlinicogemelli.it (L.R.); roberta.pietrobono@unicatt.it (R.P.); daniela.orteschi@policlinicogemelli.it (D.O.); 3 Sezione di Neuropsichiatria Infantile, Dipartimento Universitario Scienze della Vita e Sanità Pubblica, Facoltà di Medicina e Chirurgia, Università Cattolica del Sacro Cuore, 00168 Rome, Italy; elisa.musto@unicatt.it (E.M.); marika.pane@policlinicogemelli.it (M.P.); eugeniomaria.mercuri@policlinicogemelli.it (E.M.M.); 4 Unità di Neuropsichiatria Infantile, Fondazione Policlinico Universitario A. Gemelli IRCCS, 00168 Rome, Italy; 5 JC Self Research Institute, Greenwood Genetic Center, Greenwood, SC 29646, USA

**Keywords:** fragile X syndrome, Duchenne muscular dystrophy, *PPP2R5D* gene, 2p25.3 deletion, personalized medicine

## Abstract

Fragile X syndrome (FXS) is the most common form of inherited intellectual disability and autism caused by the instability of a CGG trinucleotide repeat in exon 1 of the *FMR1* gene. The co-occurrence of FXS with other genetic disorders has only been occasionally reported. Here, we describe three independent cases of FXS co-segregation with three different genetic conditions, consisting of Duchenne muscular dystrophy (DMD), *PPP2R5D*--related neurodevelopmental disorder, and 2p25.3 deletion. The co-occurrence of DMD and FXS has been reported only once in a young boy, while in an independent family two affected boys were described, the elder diagnosed with FXS and the younger with DMD. This represents the second case in which both conditions coexist in a 5-year-old boy, inherited from his heterozygous mother. The next double diagnosis had never been reported before: through exome sequencing, a girl with FXS who was of 7 years of age with macrocephaly and severe psychomotor delay was found to carry a *de novo* variant in the *PPP2R5D* gene. Finally, a maternally inherited 2p25.3 deletion associated with a decreased level of the *MYT1L* transcript, only in the patient, was observed in a 33-year-old FXS male with severe seizures compared to his mother and two sex- and age-matched controls. All of these patients represent very rare instances of genetic conditions with clinical features that can be modified by FXS and *vice versa*.

## 1. Introduction

Fragile X syndrome (FXS, OMIM #300624) is a leading monogenic cause of intellectual disability (ID), with an estimated prevalence of approximately 1:7000 males and 1:11,000 females [1]. Its incidence in males has been estimated at 1:5000 males [2]. The syndrome is almost exclusively caused by the expansion of a CGG trinucleotide repeat in the 5′ UTR of the *FMR1* gene beyond 200 units (“full mutation”, FM), followed by the DNA methylation of the promoter region (“methylated full mutation”, MFM) and *FMR1* gene silencing. This loss-of-function mutation is responsible for FXS [3]. Depending on the size of the CGG repeat, two other main classes of alleles at the *FMR1* locus may be defined: (1) normal alleles with 6–55 CGGs, with the most common alleles having 29–30 repeats; (2) premutation (PM) alleles with 56–200 CGGs [4]. The co-occurrence of FXS with other genetic conditions has been occasionally reported, as specified below. Recently, a patient with severe ID and autism was found to be affected by FXS while also carrying a pathogenic variant in *MED12* (MEDiator complex subunit 12), an X-linked gene located in Xq13.1 [5]. Five patients with both FXS and Down syndrome have been reported [6]. Furthermore, five FM female fetuses were identified by prenatal diagnosis to have the 45,X/46,XX mosaics for Turner syndrome [7]. Moreover, the association between FXS and autism spectrum disorder (ASD) is very common. In 713 FXS patients, 50% of FXS males and nearly 20% of FXS females met the diagnostic criteria for ASD. Actually, FXS is the most common known monogenic condition associated with ASD, accounting for an estimated 1% to 6% of all ASD cases [8]. However, the autistic features in FXS are not due to a double genetic cause but can instead be attributed to the variable phenotypic spectrum of the syndrome. Likewise, Wu and co-authors identified a previously unrecognized association of Duchenne muscular dystrophy (DMD) with ASD [9]. DMD (OMIM #310200) is the most common and severe form of muscular dystrophy. It is caused by pathogenic variants in the *DMD* gene located in Xp21 leading to a lack of functional dystrophin protein [10]. DMD is an X-linked recessive condition that affects around 1 in 5000 newborn boys [11]. The first symptoms usually appear at the age of 2 to 3 years, then the gradual loss of muscle tissue and function leads to wheelchair dependency at the median age of 12 years and to the need for assisted ventilation around the age of 20 years, leading to premature death, usually in the third or fourth decade of life [12]. Since the dystrophin protein 71 (Dp71) is widely expressed in the brain, learning difficulties and cognitive impairments are also prevalent in DMD patients [13]. The co-occurrence of DMD with other distinct genetic entities as a result of non-contiguous mutational events is extremely rare. Jiang and colleagues reported on a 7-year-old girl affected by DMD with multiple congenital anomalies suggestive of oculo-facio-cardio-dental (OFCD) syndrome [14]. Indeed, the girl had a frameshift variant in the *BCOR* gene (in Xp11.4), responsible for the OFCD phenotype. Similarly, a Vietnamese girl affected by DMD and carrying a deletion of exons 12–19 of the dystrophin gene was found to have a 46,XY karyotype and a variant in the androgen-receptor gene on Xp21.2, leading to male pseudohermaphroditism [15]. In addition, few cases of DMD have been documented in association with chromosome abnormalities (Down syndrome, Turner syndrome). The co-existence of DMD and FXS is anecdotal. To the best of our knowledge, only one case of atypical DMD with FXS is known [16]. Furthermore, one peculiar family is described by Todorova et al. [17], where an asymptomatic mother, carrier of three X-linked disorders, had two boys both suffering from severe ichthyosis, one also with FXS and the other one with DMD.

Recently, an autosomal dominant form of ID (OMIM #616355) was described with *de novo* heterozygous variants in the *PPP2R5D* (Protein Phosphatase 2, Regulatory subunit B (B56) Delta) gene on chromosome 6p21 [18–20]. Hypotonia, delayed psychomotor development (from moderate to severe), and poor or absent speech are common findings in these patients, while some show signs of overgrowth (macrocephaly, increased height), hydrocephalus, and seizures. Recently, an early-onset form of Parkinsonism was observed in three patients (aged between 25 and 40 years) with the p.E200K variant in the *PPP2R5D* gene [21]. The association between FXS and *PPP2R5D*-related ID has not yet been reported.

Furthermore, an additional form of autosomal dominant ID has been associated with deletions or duplications of chromosome 2p25.3 encompassing several genes, including *MYT1L* (Myelin transcription factor 1-like) (OMIM #616521). Heterozygous variants in the *MYT1L* gene are correlated to obesity, ID, seizures, speech delay, ASD, aggressive behavior, and stereotypic movements [22,23,24,25]. Mayo et al. [26] reported a girl with neonatal hypotonia, microcephaly, delayed psychomotor development, autistic features and seizures; she was not obese but had a *de novo* intragenic deletion of the *MYT1L* gene. Thus, the haploinsufficiency of *MYT1L* underlies an autosomal dominant form of ID associated with a behavioral disorder that has never been associated with FXS before.

Here, we report on three independent patients affected by FXS and an additional genetic condition. Our first patient represents the second case reported in the literature with a concomitant diagnosis of both DMD and FXS. The second patient is a girl with FXS with a pathogenic variant in the *PPP2R5D* gene, while the third patient is a boy who has FXS and is simultaneously a carrier of a maternally inherited 2p25.3 deletion with low levels of *MYT1L* expression. The association of FXS with the *PPP2R5D* variant and 2p25.3 deletion is reported for the first time.

## 2. Materials and Methods

### 2.1. Patients

Patients and their family members provided written informed consent for the molecular analysis performed in this study as well as for the sharing of clinical information. The study protocol was approved by the Ethics Committee of the Catholic University of Rome (prot. N. 9917/15 and prot.cm 10/15). DNA and RNA were extracted from peripheral blood leukocytes using standard salting-out and Trizol protocols (Thermo Fisher scientific, Waltham, MA 02451, USA), respectively. RNA was analyzed to gauge expression levels of *MYT1L* in Patient 3, his mother, and two neurotypical controls (one male aged 31 and one female aged 70).

### 2.2. Molecular Diagnosis

FXS diagnostic testing was performed through standard fluorescent PCR (GC-rich PCR system, Merck, Rome, Italy) or with the AmplideX^®^ PCR assay (Asuragen, Austin, TX, USA) in order to assess the size of the expanded CGG repeat. To evaluate the methylation status of the *FMR1* promoter MS-MLPA (Methylation Sensitive-Multiple Ligation Probe Assay), the specific assay for the *FMR1-AFF2* locus in Xq27.3 (code ME029B2, MRC Holland, 1052 Amsterdam, The Netherlands) was employed.

DMD diagnosis was confirmed using the specific MLPA assay for the *DMD* locus in Xp21.2 (code P034 and P035), following the manufacturer’s protocol (MRC Holland, Amsterdam, The Netherlands).

Array-CGH (Comparative Genomic Hybridization) analysis (Agilent Technologies 4x44K kit, Santa Clara, CA, USA) was performed following the manufacturer’s protocol.

Whole Exome Sequencing (WES) was carried out by the NextSeq 500 platform (Illumina, Cambridge, UK) after the selective enrichment of the coding sequence (exome) with the SureSelectXT2 Clinical Research Exome kit (Agilent technologies, Santa Clara, CA, USA). Target sequences were covered with the following depths: mean target region coverage of more than 60 reads/nucleotide; >95% of the region covered almost 20 times; >91% of the region covered almost 30 times. Variant calling was performed by HaplotypeCaller (v.4.0.2.1), while downstream variant filtering and prioritization required VarSeq (www.goldenhelix.com/products/VarSeq/ accessed on 15 September 2021).

### 2.3. Expression Analysis of MYT1L

An amount of 500 ng of total RNA was retro-transcribed into cDNA using the SensiFAST cDNA Synthesis kit (Bioline), according to the manufacturer’s instructions. For a relative quantification of the *MYT1L* transcript using the ABI7900HT machine (Thermo Fisher scientific), the following pre-developed TaqMan^®^ assays were employed: *MYT1L* (Hs.PT.58.4186520, IDT, location exon 19–20) and *GAPDH* (glyceraldehyde-3-phosphate-dehydrogenase) (Hs.PT.39a.22214836, IDT), the latter of which was used for normalization. The cycle parameters were 2 min at 50 °C and 10 min at 95 °C, followed by 40 cycles of 15 s at 95 °C (denaturation) and 1 min at 60 °C (annealing/extension). The relative quantification of target transcript (*MYT1L*) vs. normalizer (*GAPDH*) was calculated as follows: 2^−(Ct(*MYT1L*))-Ct(*GAPDH*))^ = 2^−ΔCt^, where ΔCt is the difference (Ct(*MYT1L*)–Ct(*GAPDH*)) and Ct is the cycle at which the detected fluorescence overcomes the threshold [27,28]. Each sample was evaluated in triplicate and three independent technical replicates were used. All variables were analyzed by means of descriptive statistics (mean, median, standard deviation, and standard error of mean) using the GraphPad Prism 7 software (La Jolla, CA, USA).

## 3. Clinical Reports

### 3.1. Patient 1

**Figure 1:**
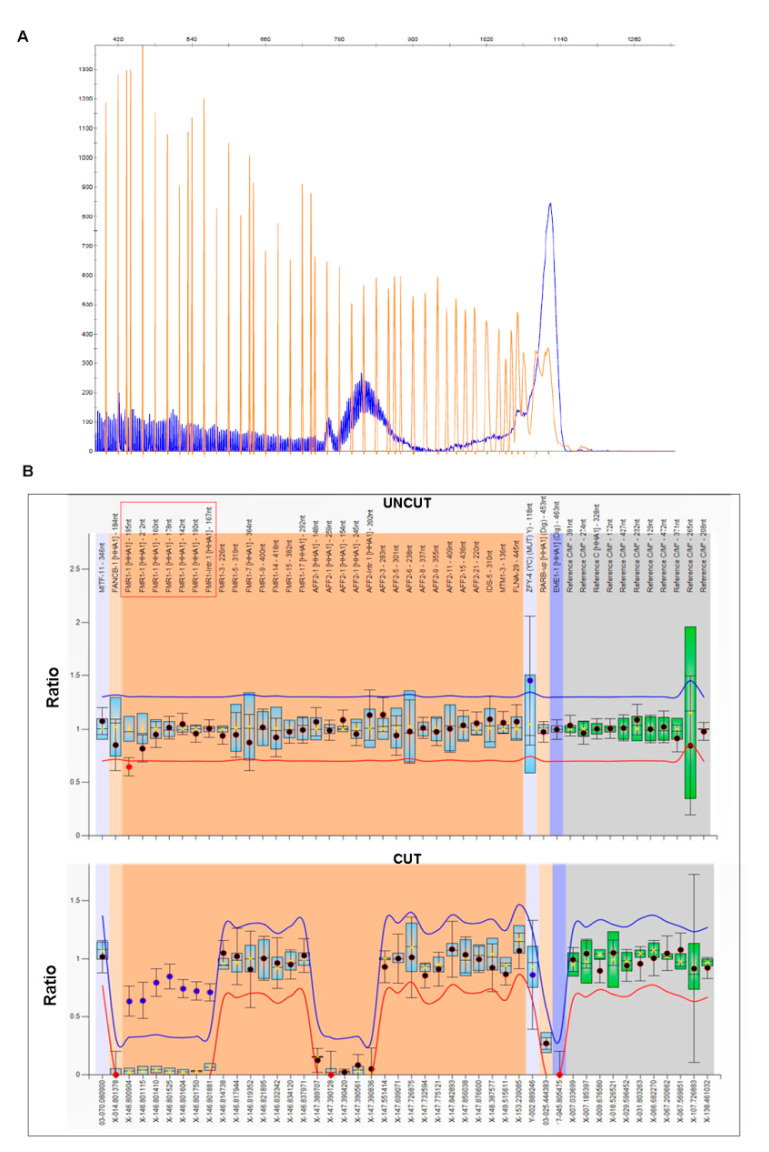
Molecular analysis of the *FMR1* locus in Patient 1. (**A**) Capillary electrophoresis of the fluorescent PCR (GC-rich PCR system) used for CGG sizing in Patient 1. He was found to carry a mosaic between a PM of approximately 170 CGGs and an FM of >200 repeats. Orange peaks represent the molecular weight marker. (**B**) Graphical representations of MS-MLPA analysis performed on Patient 1’s genomic DNA to assess DNA methylation at the *FMR1* locus. Upper panel represents the results before the digestion with methylation-sensitive *Hha*I enzyme (uncut), while the bottom panel represents the results after the cut. The *Hha*I-sensitive probes are indicated in square brackets. Note that *FMR1* probes (red box) remained uncut due to the presence of DNA methylation.

A 5-year-old boy with a molecularly confirmed diagnosis of DMD was referred for further clinical evaluation because of ID, ASD, joint hyperlaxity, and morphogenetic anomalies. A history of epilepsy with tonic–clonic seizures, photosensitivity, and moderate elevation in serum creatinine phosphokinase (CPK) levels following physical exercise was reported in his mother. He was born from non-consanguineous parents after an uneventful dizygotic twin pregnancy. A cesarean section was performed at 35 weeks of gestation due to a twin pregnancy. His birth weight was 2080 g (50th centile), and his Apgar scores were 8^1^ and 9^5^. His parents reported initial concerns during the child’s first year of life. A lack of eye contact, visual tracking, and social interest were noted from early on, associated with delayed milestones. He reached head control at 3 months and could not roll over and sit up without support till the age of 14 months; he walked independently at 4 years of age. On EEG, focal spikes over the frontal region and the left temporal region and generalized spike-and-wave complexes during sleep were detected in the absence of overt epilepsy. A brain MRI showed normal results. Following the detection of an increased CPK level (13,000 UI/L) and elevated liver enzymes (AST 272 U/L, ALT 388 U/L), muscular dystrophy was suspected at the age of 4 years and the child underwent the genetic analysis of the dystrophin gene. The deletion of exons 46-51 of the gene was detected through MLPA, consistent with the diagnosis of DMD. The mother was found to be a heterozygous carrier, as expected from her increased CPK level. When evaluated at the age of 5 years, the child presented with severe developmental delays and autistic features, including poor eye contact, the absence of protodeclarative pointing, attention deficit, and inadequate social-communicative abilities. He could not follow simple instructions and a cognitive test could not be performed. Joint hyperlaxity and peculiar facial traits were noted, including a high forehead, epicanthic folds, deep-set eyes, an elongated face, and large ears. Notwithstanding the reported high rate of cognitive impairment and neurobehavioral abnormalities in DMD, the complexity of the whole clinical phenotype of our patient—in particular, the association of motor delay and severe ID with ASD and the distinctive facial dysmorphisms—led us to hypothesize comorbidity with an additional genetic condition, namely, with FXS. Following array-CGH analysis, which detected no additional CNVs, except for the deletion formerly identified in the dystrophin gene, the child underwent *FMR1* molecular analysis. He was found to be a mosaic carrier of a large premutation (PM) with 170 CGGs and of an MFM with an expansion above 200 CGG triplets, confirming the clinical hypothesis of FXS (Figure 1). His unaffected mother was heterozygous for a normal allele with 20 CGGs and a premutation allele with 80 triplets. The boy is currently receiving a rehabilitation program with slight improvement shown, especially in his motor skills.

### 3.2. Patient 2

**Figure 2:**
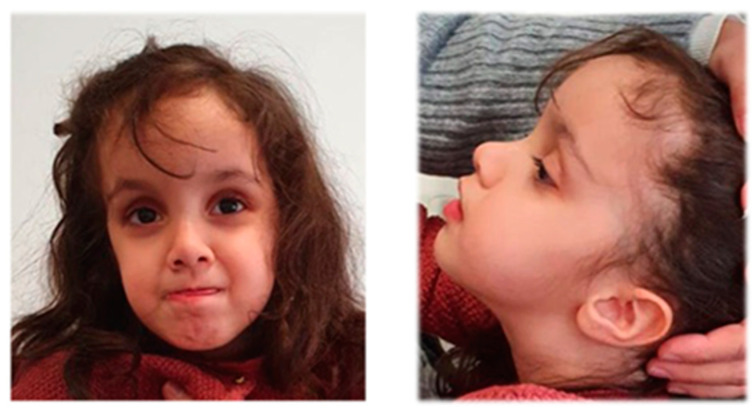
Patient 2 at 5 years and 5 months old. Note the broad and rounded forehead with frontal bossing, small nose with low nasal bridge and anteverted nostrils, sparse eyebrows in the medial portion, and hypotonic gestalt.

This 7-year-old girl is the only child of non-consanguineous parents. She was born at 39 weeks of gestational age via an urgent cesarean section due to maternal premature rupture of the membranes (PROM). At birth, she presented respiratory distress and her Apgar scores were 5^1^ and 8^5^. Her birth weight was 3550 g (75th centile), her length was 53 cm (90th centile), and her OFC 36.5 cm (around 98th centile). Soon after birth, she developed spontaneous tremors of the upper limbs, axial hypotonia, and apnea episodes treated with phenobarbital and oxygen, respectively. A brain ultrasound and MRI were normal. EEG displayed continuous activity, with occasional sharp elements in the right temporo-occipital area. Audiometric and fundus oculi examinations were both normal. She reached head control at 3.5 months, sitting position at 10 months, and non-autonomous standing station at 15 months. Up to 15 months, she presented difficulties in handling objects with coarse grip. Language was poor with very few words developed at 13 months. Psychomotor delay was accompanied by macrocephaly: until 4 months of age, OFC was at the 98th centile and from 8 to 10 months it was abundantly above the 98th centile. Upon physical examination, she presented with a broad and rounded forehead, a small nose with saddle root and anteverted nostrils, a reverse epicanthus, sparse eyebrows in the medial portion, fetal finger pads, ligamentous hyperlaxity, and a sandal gap with prominent heel (Figure 2). Upon clinical evaluation performed at 4 years and 5 months of age, the young girl pronounced few simple words, walked with a broad-based gait and showed a lack of sphincter control. Negative results came from the direct nucleotide sequencing analysis of the following genes: lamin A/C, *SEPN1*, *NFIX*, *EZH2*, *NSD1*, *SETD2*, *COL6A1*, *COL6A2,* and *COL6A3*. Array-CGH revealed a “likely benign” 9q21.31 duplication of approximately 200 kb, with no associated genes https://www.deciphergenomics.org/ accessed on 18 January 2021 and http://dgv.tcag.ca/dgv/app/home accessed on 18 January 2021). Parental origin was not investigated. Molecular analysis for FXS revealed heterozygosity for a normal allele of 29 CGG repeats and a series of expanded alleles in the range of PM and FM (between 73 and >200 CGGs). After the diagnosis of FXS in the proband was established, the parents were also examined with the following results: her father carried the 29 CGG allele, while her mother had a normal allele of 23 triplets and a PM of 121–131 CGG triplets. As the diagnosis of fragile X syndrome could not explain all the clinical findings of the proband, a trio WES was undertaken and revealed the presence in the proband of a *de novo* heterozygous variant c.592G>A p.(Glu198Lys) in the *PPP2R5D* gene (NM_006245.3). This variant is reported in HGMD (Human Gene Mutation Database; CM153575) [18] and never in GnomAD (Genome Aggregation Database). The same variant was previously reported *de novo* in patients affected by moderate/severe ID [18,19].

### 3.3. Patient 3

**Figure 3:**
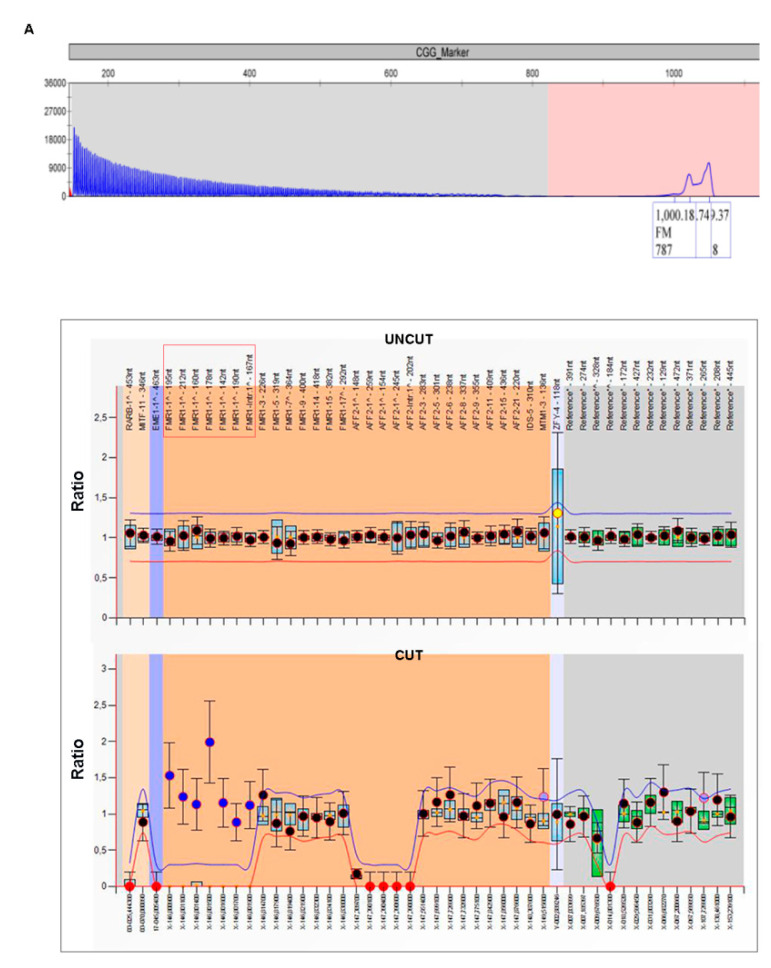

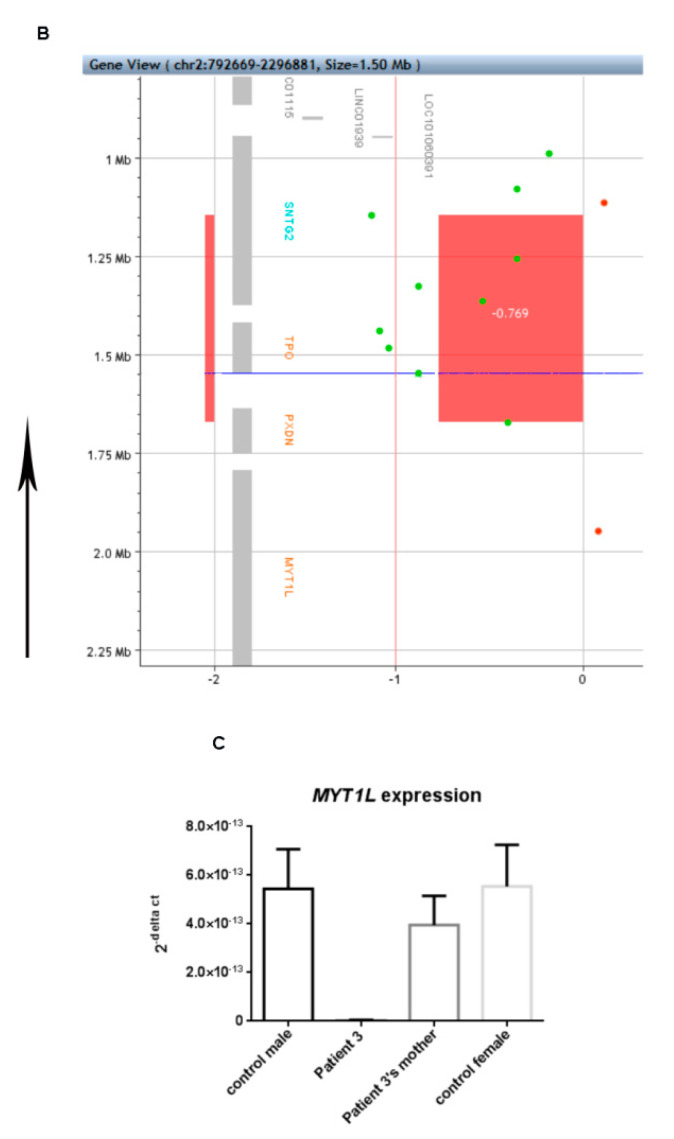
Results of molecular analyses performed in Patient 3. (**A**) Capillary electrophoresis of the fluorescent PCR (AmplideX® PCR assay, Asuragen) used for CGG sizing in Patient 3 (upper panel). He presented an FM of >200 repeats. MS-MLPA analysis at the *FMR1* locus performed on Patient 1’s genomic DNA showed methylation status of the FM. Middle panel represents the results before the digestion with methylation-sensitive *Hha*I enzyme (uncut), while the bottom panel represents the results after the cut. *FMR1* probes (red box) remained uncut due to the presence of DNA methylation. (**B**) Array-CGH analysis revealed the extension of the 2p25.3 microdeletion. The deleted region spans more than 500 kb, encompassing the *SNTG2*, *TPO*, and *PXDN* genes, and likely *MYT1L*. The deletion has apparently the same extension in the proband and in his mother (using an Agilent 44K array). The arrow indicates the sense of *MYT1L* transcription. (**C**) Relative quantification of *MYT1L* transcript through real-time PCR on peripheral blood leukocytes of the proband, his mother, and two neurotypical controls (one male and one female). Levels of *MYT1L* RNA were reduced in the proband compared to those of his mother and neurotypical controls. Values reported on the y-axis represent relative transcriptional levels normalized to transcript *GAPDH* (2^-delta ct^). Histograms represent the mean value of three independent technical replicates; bars represent the standard error of the mean (SEM).

Patient 3 is a 33-year-old man who is the second child of non-consanguineous parents. He started walking and saying his first words at the age of 2. At the age of 10 years he suffered his first seizure episodes and when he was 22 years old he had a coma episode following a severe seizure crisis. Brain angio-MRI showed temporo-mesial sclerosis, left A1 segment agenesis with origin of the left anterior vertebral artery from the right circle, asymmetry of the supratentorial ventricular system due to the prevalence of the right trigone, and an occipital horn. He is currently still undergoing anticonvulsive treatment with valproic acid, oxcarbazepine, and topiramate. His character is calm, with a few nervous jerks (due to seizure medication). Presently, he attends a day center and practices sport (judo) and recreational activities (dancing). Family history revealed two further male patients (both sons of a maternal cousin) affected by ID of an unknown cause and diagnosis. Physical examination showed an elongated face, high forehead, wide and anteverted ears, a long and flat philtrum, midface hypoplasia, joint hyperlaxity, and hypotonia. A molecular analysis of the *FMR1* gene and array-CGH were performed, revealing an *FMR1* MFM allele (>200 CGGs) (Figure 3A). Following this result, his mother was tested and found to be heterozygous for a normal allele of 30 CGG triplets and a PM allele with 79 CGGs. She underwent menopause at 39 years and had a spontaneous fracture of the femur at 50 years. Computerized bone mineralometry showed severe osteoporosis. No history of seizures was reported in the mother. The proband’s sister was found to carry a PM of the *FMR1* gene and she had a first unaffected daughter and a second son affected by FXS. Additionally, array-CGH revealed a chromosome 2p25.3 deletion spanning around 500 kb, again derived from his premutated mother (Figure 3B). The deleted region on chromosome 2 was included between positions 1,145,059 and 1,670,349 (according to Genome Browser Assembly hg19, GRCh37, February 2009) and encompasses the *SNTG2*, *TPO*, and *PXDN* genes, and possibly *MYT1L*. There were no specific probes in this last locus at the 44 K resolution of the employed array. Due to the association of the *MYT1L* gene with ID and seizures and since the clinical presentation of the proband was complicated by severe convulsions, we quantified the *MYT1L* transcript levels in the proband and his mother in order to verify if there was a “positional effect” of the microdeletion on chromosome 2. The results of real-time PCR revealed a decreased level of *MYT1L* transcript in the proband when compared to his mother and neurotypical controls (Figure 3C).

## 4. Discussion

Clinical heterogeneity underlies many genetic conditions due to the contribution of environmental factors as well as modifier genes. Atypical clinical presentation caused by the co-occurrence of two monofactorial diseases is apparently a rare event. However, an increasing number of double genetic diagnoses are being detected using whole-exome and whole-genome sequencing. Depending on the precise genetic diagnosis, familial recurrence can change and the diagnostic procedures used for prenatal genetic testing can be different as well.

Our report deals with the co-occurrence of FXS and a second genetic condition in three unrelated subjects. One male patient was first diagnosed with DMD caused by the deletion of exons 46-51 of the dystrophin gene of maternal origin. One year later, he was referred because of unexplained ASD with severe ID, facial dysmorphisms and joint hyperlaxity. All these signs prompted us to check for the *FMR1* full mutation, with positive results. He represents the second patient in the medical literature with a concurrent diagnosis of DMD and FXS. The first such patient was reported by Natori et al. [16], who described a 9-year-old boy with DMD whose clinical phenotype overlapped with FXS. Another paper described the independent segregation of FXS and of DMD in two different brothers with the same mother, who apparently carried fragile X on one of her X chromosomes and DMD on the other X chromosome [17]. On the other hand, in Patient 1, a double event occurred in the carrier mother—namely, an intragenic deletion in the dystrophin gene and an expansion of the CGG repeat in the *FMR1* gene. These two mutational events were completely distinct and happened independently in two genes that were located far from each other on the opposite sides of the same X chromosome.

The second female patient received a first diagnosis of FXS associated with a maternally inherited full mutation in *FMR1*. However, she presented with an atypical phenotype, including severe neurodevelopmental disability, macrocephaly, and infantile hypotonia. By WES, a *de novo* heterozygous variant in *PPP2R5D* was detected, fully concordant with her clinical presentation. The *PPP2R5D* gene maps onto chromosome 6 and codes for a regulatory subunit of a phosphatase complex involved in cell cycle control and in the PI3K-AKT pathway [20]. The association between FXS and autosomal dominant ID caused by *PPP2R5D* variants has never been reported before.

Finally, our third patient presented a classical FXS phenotype whose only discordant note was the severity of his epilepsy. Convulsions in FXS patients are usually responsive to treatment and improve with growth, unlike in Patient 3. Indeed, seizures are present in around 40% of FXS cases and are usually partial, regress with time, and are amenable to treatment [29]. Hypersensitivity to sensory stimuli, especially audiogenic ones, and hyperarousal are also frequently found in FXS patients [30]. The microdeletion on chromosome 2 in the patient and his mother did not include the *MYT1L* gene, which is associated with an autosomal dominant form of ID and seizure (OMIM #616521), though the resolution of the diagnostic array-CGH employed cannot exclude this possibility. The quantification of the *MYT1L* transcript showed a reduction in the proband but not in his mother, who did not have any history of seizures. A possible explanation for the clinical discrepancy between the unaffected mother and her son, apparently both carriers of the same 2p25.3 microdeletion, could be due to the meiotic amplification of a smaller deletion present in the mother, as already described by [31].

## 5. Conclusions

The three patients described here with a concomitant diagnosis of FXS and another genetic condition (Duchenne muscular dystrophy, *PPP2R5D*-related and *MYT1L*-related intellectual disability, respectively) underline the importance of a thorough clinical evaluation in order to select the most appropriate genetic test. The combination of exome- or even genome-wide techniques with the “deep phenotyping” provided by experienced clinical geneticists will allow the dissection of overlapping phenotypes. Unusual and rare cases whose clinical findings do not entirely fit within the typical FXS phenotype should prompt further investigations in order to detect possible co-occurring conditions, rather than stopping at the first genetic diagnosis and attributing the discrepant findings to variable expression.

## Data Availability

The authors confirm that the data supporting the findings of this study are available within the article.
